# Conventional Anthelmintic Concentration of Deltamethrin Immersion Disorder in the Gill Immune Responses of Crucian Carp

**DOI:** 10.3390/toxics11090743

**Published:** 2023-08-31

**Authors:** Hao Wu, Xiping Yuan, Jinwei Gao, Min Xie, Xing Tian, Zhenzhen Xiong, Rui Song, Zhonggui Xie, Dongsheng Ou

**Affiliations:** Hunan Fisheries Science Institute, Changsha 410153, China; wh17380133463@163.com (H.W.); kerryuan@163.com (X.Y.); gaojinwei163@163.com (J.G.); xieminhaha@126.com (M.X.); tianx2323@163.com (X.T.); 13763092902@163.com (Z.X.); xieice123@163.com (Z.X.); njdodx@163.com (D.O.)

**Keywords:** deltamethrin, gill barrier, TMT-based proteomics, gill microbiota, cell apoptosis

## Abstract

Current treatment strategies for parasitic infectious diseases in crucian carp primarily rely on chemotherapy. As a commonly used antiparasitic agent, deltamethrin (DEL) may have the potential adverse effects on external mucosa of fish such as gills. In this study, 180 healthy juvenile crucian carp (*Carassius auratus*) (average weight: 8.8 ± 1.0 g) were randomly divided into three groups for 28 days, which were immersed in 0 μg/L, 0.3 μg/L, and 0.6 μg/L of DEL, respectively. The results of histological analysis revealed that severe hyperplasia in the secondary lamellae of gills was observed, and the number of goblet (mucus-secreting) cells increased significantly after DEL immersion. TUNEL staining indicated that the number of apoptotic cells increased in crucian carp gill. At the molecular level, the mRNA expression analysis revealed significant upregulation of apoptosis (caspase 3, caspase 8, and bax), autophagy (atg5 and beclin-1), and immune response (lzm, muc5, il-6, il-8, il-10, tnfα, ifnγ, tgfβ, tlr4, myd88, and nf-kb), whereas tight junction-related genes (*occludin* and *claudin12*) were downregulated after DEL immersion, suggesting that DEL immersion altered innate immunity responses and promoted mucus secretion. Moreover, tandem mass tag (TMT)-based proteomics revealed that a total of 428 differentially expressed proteins (DEPs) contained 341 upregulated DEPs and 87 downregulated DEPs with function annotation were identified between the control and DEL groups. Functional analyses revealed that the DEPs were enriched in apoptotic process, phagosome, and lysosome pathways. Additionally, DEL immersion also drove gill microbiota to dysbiosis and an increase in potentially harmful bacteria such as *Flavobacterium*. Overall, this study showed that DEL elicited shifts in the immune response and changes in the surface microbiota of fish. These results provide new perspectives on the conventional anthelmintic concentration of DEL immersion disorder of the gill immune microenvironment in crucian carp and theoretical support for future optimization of their practical application.

## 1. Introduction

The proliferation of intensive aquaculture has led to the emergence of parasitic outbreaks in aquatic animals, which frequently result in mortality and diminished yields [1,2]. Insecticides are widely employed as a treatment for ectoparasitic infections; however, their excessive utilization has raised persistent concerns. Deltamethrin (DEL) belongs to the category of type II pyrethroid insecticides and is capable of efficiently managing or averting parasitic diseases in aquaculture by disrupting the regular nervous system functioning of ectoparasites [3,4,5]. Presently, DEL has gained widespread acceptance as the preferred method for eliminating monogeneans, parasitic organisms that predominantly inhabit the external surface of fish. Consequently, DEL has emerged as a viable substitute for organophosphate and organochlorine insecticides [5,6].

Typically, immersion is the preferred method for administering DEL to fish, resulting in direct exposure of the exogenous compound to the mucosal tissues of fish, particularly in the external mucosa [7]. While DEL is generally regarded as safe, overuse can result in significant harm to the mucosal tissues of farmed animals and substantial economic losses [8]. The industry refers to this occurrence as aquatic drug poisoning. Simultaneously, DEL possesses lipophilic properties that facilitate its entry into organisms and subsequent penetration of internal organs via the gill and intestine [7,9]. After DEL misuse and overdose after administration, mortality and sublethal effects on aquatic organisms including oxidative impairment, neurotoxic responses, immunotoxic effects, and gut microbiota dysbiosis have been observed [10,11,12,13,14,15]. Indeed, the aquaculture industry frequently employs a conventional anthelmintic concentration of DEL within the range of 0.3 to 0.6 μg/L [16,17,18]. Notwithstanding the evidence previously obtained, which suggests that chronic exposure to low doses of DEL has a disruptive effect on both intestinal health and the intestinal microbiota [12], insufficient data exist regarding the negative impacts of prolonged exposure to DEL at therapeutic levels on the external mucosal tissue of aquatic organisms.

Based on our current understanding, the gill serves as a crucial organ in fish, responsible for regulating osmosis, excreting ammonia, facilitating oxygen uptake, and performing detoxification functions [19,20,21,22]. Moreover, due to their status as the primary point of contact between organisms and xenobiotics, gills possess a histological structure that is relatively fragile, consisting of simple epithelial cells. As a result, they are highly susceptible to the deleterious effects of waterborne pollution and are particularly vulnerable to damage caused by pesticides [23,24]. There is a wealth of research indicating that exposure to pesticides can trigger oxidative stress, inflammation, autophagy, and apoptosis in the gills of fish [25,26,27]. Feng et al. found that the administration of abamectin to carp resulted in the induction of oxidative stress, inflammation, and apoptosis, while also inhibiting autophagy through the PI3K/AKT/mTOR signaling pathway [28]. Sun et al. demonstrated that 4-octylphenol caused inflammatory injury and immunosuppression via tlr7/IκBα/NF-κB pathway in gill of common carp [29]. In turn, the inflammation of fish gill epithelia results in heightened permeability to pesticides, thereby exacerbating gill impairment [30]. The maintenance of gill barrier function, facilitated by mucin and tight junction proteins, is imperative in preventing the infiltration of detrimental agents into the organism through the epithelium [31]. When the concentration of xenobiotics surpasses the typical threshold that cells can endure, discernible histological alterations and physiological dysfunction may occur in the gill, leading to cellular demise and tissue harm [32]. Additionally, gill mucosal surfaces are enveloped by a layer of mucus that necessitates a diverse range of commensal microbial colonization. Notably, recent years have witnessed novel revelations regarding the microbiota of fish mucosal surfaces [19,33,34]. Furthermore, the gill microbiome plays a role in mitigating host stress in response to external environmental factors, in conjunction with the gills [33,35,36]. Lai et al. demonstrated that the gill microbiota of marine medaka could respond to osmotic stress, and the dominant microbiota including *Vibrio*, *Pseudomonas*, and *Cetobacterium* were found to change the glycosaminoglycan and chitin metabolisms [35]. In recent years, an increasing number of studies have endeavored to utilize multi-omics data to elucidate the underlying factors contributing to gill tissue toxicity. Transcriptomic and proteomic analyses have demonstrated the immune deficiencies in the gill of crucian carp in response to *Aeromonas hydrophila* [37]. The utilization of transcriptomics and metabolomics methodologies unveiled the gill toxicity of crucian carp induced by DEHP [21]. The integration of transcriptomics and metabolomics has revealed the manifestation of gill damage in common carp (*Cyprinus carpio*) following exposure to nanosilver [38]. Nonetheless, there is no extant research demonstrating gill impairment in fish through the application of integrated omics following DEL immersion. 

The therapeutic use of DEL immersion is intended to manage various diseases caused by parasites in the gills of farmed crucian fish. The crucian carp (*Carassius auratus*), being a significant economic species, has been proven to be highly responsive when investigating the detrimental impacts of pollutants on aquatic organisms. However, there is limited knowledge on the impact of prolonged exposure to DEL on the commensal microbiome and gill injury of early-stage crucian carp. Herein, in this study, we aimed to (a) investigate the detrimental impacts of chronic DEL exposure on the histological, mucosal barrier, inflammation, and apoptosis of the gill of crucian carp and (b) elucidate the differential expression profiling of proteins and the fluctuation of commensal microorganisms in the gill mucosa through the application of proteomic and 16S rDNA sequencing techniques.

## 2. Material and Methods

### 2.1. Ethical Statement

The experiment procedures (license no. HNFI20210322) in this study were approved by the Institutional Animal Use in the Research Committee of Hunan Fisheries Science Institute (procedure approval 22 March 2021).

### 2.2. Chemicals

Pharmaceutical-grade DEL (CAS: 52918–63-5, purity ≥ 98%) was procured from Aladdin (Shanghai, China) and subsequently dissolved in acetone (purity ≥ 98%). The acetone used was of analytical grade and was purchased from Aldrich (St. Louis, MO, USA). The resulting sample solution was adjusted to a concentration of 10.0 μg/L. The DEL stock solution was then stored in a brown bottle at 4 °C for future use.

### 2.3. Experimental Design and Sampling

The healthy juvenile crucian carp (*Carassius auratus*), with a mean weight of 8.8 ± 1.0 g and mean body length of 6.9 ± 0.4 cm, were procured from Hunan Fisheries Science Institute (Changsha, China). Subsequently, they were reared under laboratory conditions for a period of two weeks to eliminate the impact of other unfavorable factors. During this period, all fish were fed with commercial feed twice a day at 8:00 and 17:00, and any remaining feed and feces were extracted via siphoning. About one-third of the water was exchanged once a day and the water quality (dissolved oxygen 7.0 ± 1.0 mg/L, temperature 25 ± 1 °C, total ammonia nitrogen 0.03 ± 0.01 mg/L) was monitored daily. Following a period of acclimation, a total of 180 fish were randomly allocated into three distinct groups, namely the control group (Con), the 0.3 μg/L DEL exposure group (DEL-L), and the 0.6 μg/L DEL exposure group (DEL-H). Each group comprised three replicated tanks, each of which contained 20 crucian carps in a 65 L plastic aquarium. Each tank was aerated continuously with an aeration stone and the water temperature was kept at 25 ± 2 °C, and the pH value was maintained at 7 with the dissolved oxygen of 7.0 mg/L. Photoperiod was maintained with a normal dark/light cycle. The determination of the DEL-L concentration was based on the therapeutic dose commonly utilized for DEL [12], while the DEL-H concentration was established by adopting 1/10 of the 96 h LC50 (safe concentration), as per the results of our acute toxicity test [11]. During the exposure trial, the fish were subjected to the original laboratory conditions for a period of 28 days. The concentrations of DEL were maintained within 5% of the target concentrations, and half of the water was renewed once a day by introducing a solution containing the same concentration of deltamethrin to maintain the predetermined level. The concentration of the exposure solution was determined using GCMS techniques at the FOREGENE company in China. All fish were fed commercial diet (crude protein 39.5%, crude lipid 7.8%) to apparent satiation twice daily (9:00 and 16:00).

All the fish were fasted on day 27 and sample collection was started 24 h after fasting. In each tank, fish were expeditiously weighed and then anesthetized using MS-222 (100 mg/L, Sigma, Kanagawa, Japan) [13]. The second and third gill arches of fish were excised, rapidly frozen in liquid nitrogen for 3 h, and subsequently stored at −80 °C for qPCR assay. Additionally, six gill samples of the Con and only DEL-H group were selected for TMT-based proteomic and 16S rDNA sequencing. Simultaneously, the second gill arch samples utilized for histopathological analyses and TUNEL assay were fixed in a 4% paraformaldehyde solution for a period of one day. 

### 2.4. Histopathological Analyses

The gill samples obtained from each group underwent a washing process with 0.7% normal saline solution to eliminate any blood stains, followed by fixation of the tissues in 4% paraformaldehyde for a duration of 24 h. Subsequently, the fixed tissues were subjected to dehydration in a series of graded ethanol, followed by clearance in xylene, embedding in paraffin wax, and sectioning into 6 μm slices utilizing a microtome (Krumdieck, Birmingham, AL, USA). Hematoxylin–eosin (HE) and alcian blue and schiff (combined AB-PAS) staining were performed for histopathological observation using commercial kits and photographed using light microscope (E200MVR, Nikon, Tokyo, Japan). Importantly, a random selection of ten complete slices from each group was utilized to calculate morphological indicators according to our previous work [11], such as the mucous cell count, through the utilization of Image-Pro Plus 6.0 software. The assessment of the mucin type present in the mucous cells was conducted, with acidic mucous cells exhibiting a blue stain and neutral mucous cells displaying a magenta stain.

### 2.5. Determination of Gills Filaments Apoptosis

The terminal deoxynucleotidyl transferase-mediated uridine 5′-triphosphate-biotin nick end-labeling (TUNEL) assay was executed utilizing a commercially available kit (Cat No. 11684817910, Roche, Basel, Switzerland) according to our previous study [13]. In brief, each second gill arch was fixed using a 4% paraformaldehyde solution and subsequently sectioned. The sections underwent a dewaxing process utilizing xylene, followed by dehydration with a gradient alcohol series, and the sections were subsequently immersed in PBS (pH 7.4) and subjected to three rounds of agitation on a decolorizing shaker. Subsequently, the sections were subjected to a 30 min incubation period in a blocking buffer containing 3% goat serum. Following this, the sections were exposed to TdT and dUTP at a ratio of 1:9 and incubated overnight at 4 °C. The sections were subjected to dropwise addition of DAPI solution, followed by an incubation period of 10 min at room temperature in the absence of light. The sections were washed thrice with PBS (pH 7.4), followed by sealing with an antifluorescence quencher (Cat No. 0100-01, Southern Biotech, Birmingham, AL, USA). The staining effect was observed using a fluorescence microscope (Nikon ECLIPSE, Japan). Finally, the average number of normal and apoptotic cells (×15 magnification) in the gill filament from each group was counted in five different fields of view from five slices using ImageJ software, and apoptosis rate was quantified as the ratio of the number of apoptotic cells to the number of normal cells.

### 2.6. Quantitative Real-Time Polymerase Chain Reaction (qRT-PCR) 

For the purpose of detecting apoptosis- and immune-related gene expression, a total of five gills was pooled from each tank and subjected to qPCR assay. The gill sample’s total RNA was extracted using the Animal Total RNA Isolation Kit (Foregene, Chengdu, China), and its quality and concentration were assessed using 1% agarose gel electrophoresis and a Nanodrop 2000 spectrophotometer (Nanodrop Technologies, Wilmington, DE, USA). The reverse transcription of the mRNA was executed in accordance with the guidelines provided by the manufacturer of the cDNA reverse transcription kit (TaKaRa, Kusatsu, Japan). The ABI 7500 fast real-time PCR system (Applied Biosystems, Waltham, MA, USA) was utilized to conduct qRT-PCR. The primer sequence of the genes is documented in Table S1. The β-actin gene was considered a housekeeping gene that standardized the relative expression of target genes using the 2^−ΔΔCT^ method.

### 2.7. TMT-Based Proteomic Analysis

The frozen gill samples underwent lysis using 300 μL of lysis buffer, which was supplemented with 1 mM PMSF. The total proteins were subsequently extracted through the utilization of sonication methodology. Following centrifugation at 12,000 rpm for 10 min, the final supernatants were gathered, and the protein concentration was assessed using the BCA assay. Following trypsinolysis, the proteins underwent TMT labeling in accordance with established protocols. Briefly, the lyophilized samples were reconstituted in 50 μL of 100 mM TEAB, and 40 μL of each sample was subsequently transferred for labeling. A quantity of 88 μL anhydrous acetonitrile was added to the TMT reagent vial at ambient temperature. The reagents were subjected to centrifugation, followed by dissolution for a duration of 5 min. Subsequently, 41 μL of the TMT label reagent was introduced to each sample to facilitate homogenization. The tubes were subjected to incubation at ambient temperature for a duration of 1 h. Subsequently, an addition of 8 μL of 5% hydroxylamine was made to each sample, followed by an incubation period of 15 min to halt the reaction. Reversed-phase chromatography fractionation of the TMT-labeled proteins was performed. The protein fractions were subjected to analysis via liquid chromatography with tandem mass spectrometry (LC-MS/MS), which was performed by OE Biotech Co., Ltd. (Shanghai, China). TMT-6-plex was selected for protein quantification method.

The bioinformatic analyses were performed in R package software. Proteome Discoverer (Version 2.4.1.15, ThermoFisher Scientific, Waltham, MA, USA) was used for protein mapping. Identifications of peptides were filtered at a false discovery rate (FDR) of 1%. Furthermore, our search for differentially expressed proteins (DEPs) was limited to fold change ratios greater than 1.20 or less than 0.83 with a *p*-value 0.05. Principal component analysis (PCA), heatmap clustering analysis, and volcano plot were generated using online tools (https://cloud.oebiotech.cn/task/ (accessed on 8 June 2023)). Kyoto Encyclopedia of Genes and Genomes (KEGG) (http://www.genome.jp/kegg/ (accessed on 8 June 2023)) and Gene Ontology (GO) (http://www.geneontology.org (accessed on 8 June 2023)) terms were analyzed using the hypergeometric distribution in order to compare DEG enrichment relative to all genes in those pathways and terms. Additionally, protein–protein interaction (PPI) networks were constructed using the STRING database. 

### 2.8. 16S rRNA Sequencing

The six gills from each tank were pooled together and each group had six biological replicates for 16S rRNA sequencing. Bacterial DNA was isolated from the gill sample using a DNeasy PowerSoil kit according to the manufacturer’s instructions (Qiagen, Hilden, Germany). The concentration and integrity of extracted genomic DNA was detected using NanoDrop 2000 spectrophotometer (Thermo Fisher Scientific, Waltham, MA, USA) and agarose gel electrophoresis. Subsequently, the bacterial 16S rRNA V3-V4 region was amplified using universal primer pairs (343F: TACGGRAGGCAGCAG; 798R: AGGGTATCTAATCCT). Next, the PCR product was sequenced using the Illumina NovaSeq6000 with two paired-end read cycles of 250 bases each. Paired-end reads were preprocessed and filtered at low quality using DADA2 with QIIME2. And the representative reads were clustered into amplicon sequence variants (ASVs) and then annotated and blasted using q2-feature-classifier. The alpha diversity indices (including Chao, Shannon, and Simpson) and the beta-diversity was calculated using QIIME software. A one-way ANOVA was utilized to examine the variations in the relative abundances at the phylum and genus levels between the DEL group and control group. The present study utilized linear discriminant analysis (LDA) effect size (LEfSe) analysis to identify microbial signatures. The LDA threshold score was set to 4 and the *p* values were set at 0.05. 16S rRNA gene amplicon sequencing and analysis were conducted by OE Biotech Co., Ltd. (Shanghai, China). In addition, the Spearman correlation was utilized to calculate the correlation analysis between the genus level of gill microbiota and the associated key DEPs via R software v3.6.3.

### 2.9. Statistical Analyses

All data are presented as mean ± standard deviation (SD). The tests employed to assess the homogeneity of variances and normal distributions were Levene’s test and the Shapiro–Wilk test, respectively. One-way analysis of variance (ANOVA) or Kruskal–Wallis test was performed to determine the significant difference in the mucous cells number, the apoptosis rate, the gene expression levels, and the microbial alpha diversity and abundance between two groups with GraphPad Prism 8.0 (GraphPad Software Inc., San Diego, CA, USA). The significance level of 0.05 (*p* < 0.05) was used. 

## 3. Results 

### 3.1. Histoarchitectural Changes and TUNEL Assay

**Figure 1:**
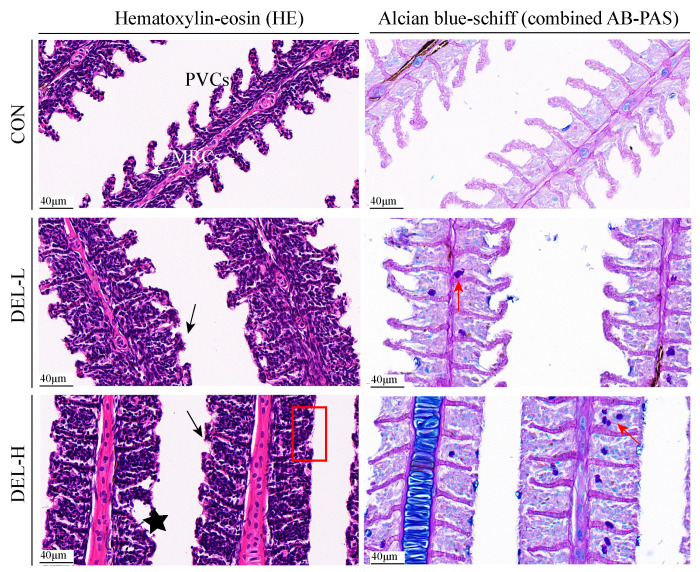
Histological examination using HE and AB-PAS staining of gill from the control, DEL-L, and DEL-H groups. MRCs: mitochondria-rich cells; PVCs: pavement cells. Red arrow: goblet (mucus-secreting) cells; black arrow: severe hyperplasia in secondary lamellae; red rectangle: gill distortion and fusion of adjacent secondary lamella; black star: epithelial cell desquamation and necrosis.

As shown in Figure 1, the changes in morphology and mucus distribution of the gills were evaluated using HE and combined AB-PAS stained sections. In this study, the gills of the control fish did not exhibit any histopathological alterations. The gill filament exhibited a relatively intact structure, with gill lamellae arranged in parallel on either side of the filament, and nuclear membranes of pavement cells (PVCs) were clear with normal morphology. The distribution of mitochondria-rich cells (MRCs) was predominantly observed at the base of the gill lamella, exhibiting an ovoid morphology. However, prolonged DEL immersion resulted in a slight disturbance to gill epithelial integrity. Notably, severe hyperplasia in the secondary lamellae was observed as the most prevalent alteration across all DEL dosages, and hyperplasia led to gill distortion and fusion of adjacent secondary lamella following DEL immersion, especially in fish of the DEL-H group. In addition, it was observed that certain instances of desquamation and necrosis occurred in the apical region of secondary lamellae in the DEL group. Meanwhile, mucous cells were unevenly distributed on the primary gill filaments and the number of goblet (mucus-secreting) cells increased significantly in the fish gills with the increase in concentration of DEL (*p* < 0.05), with the cell number peaking in the gill in the DEL-H group. In the present study, a significant proportion of neutral mucin-containing mucous cells were observed, while sporadic presence of acid mucin was detected in the gill samples.

Detailed information on goblet cell numbers are shown in Supplementary Materials (Figure S1).

**Figure 2:**
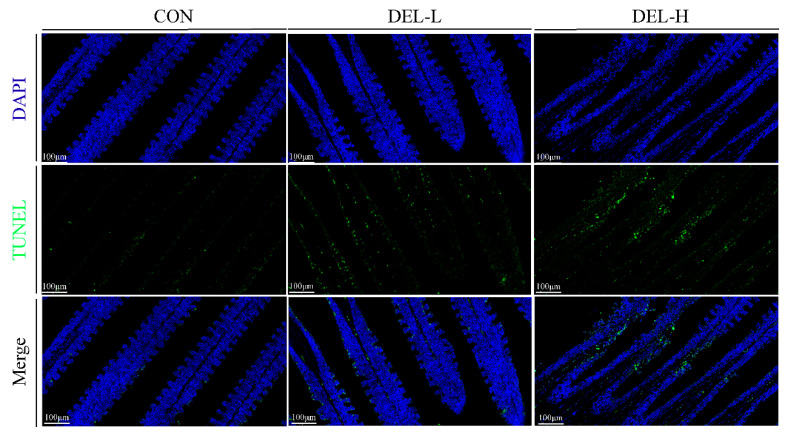
TUNEL staining of gill tissue. The apoptotic cells on the tissue sections showed green fluorescence; blue represents DAPI nuclear staining.

Further, in order to better evaluate the degree of gill cell damage following DEL immersion, we measured the ratio of apoptotic cells in each group by averaging the number of positive TUNEL signals, and the results demonstrate that immersion with DEL significantly increased the ratio of apoptotic cells in the gill of crucian carp, as compared to the control group (*p* < 0.05, Figure 2). It is noteworthy that the rates of apoptosis positivity exhibited a significant dose-dependent effect, as shown in Figure S2.

### 3.2. Gene Expression Analysis 

#### 3.2.1. Expression of Tight Junction-Related Genes 

**Figure 3:**
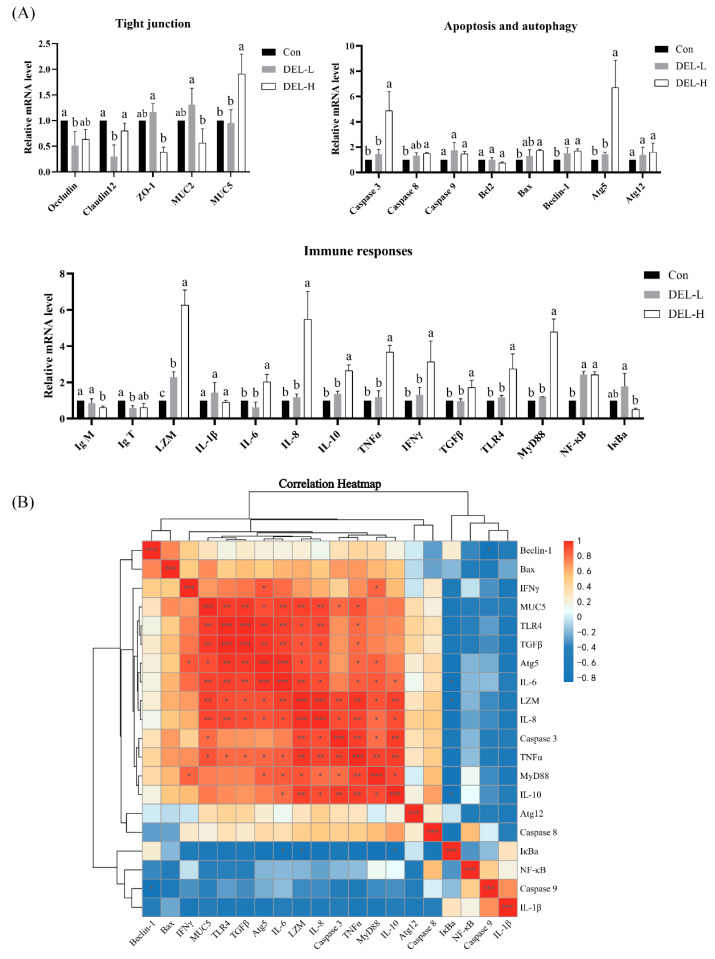
(**A**) The mRNA levels of tight junction, apoptosis, autophagy, and immune-related genes of gill from the control, DEL-L, and DEL-H groups. The values are expressed as the means ± SDs (*n* = 5). Different letters represent statistically significant differences among the experimental and control groups (*p* < 0.05). (**B**) Pearson correlations between gene expression were visualized. * represent *p* < 0.05, ** represent *p* < 0.01, *** represent *p* < 0.001.

To analyze the effects of immersion with DEL on tight junction, the mRNA levels of *occludin*, *claudin12*, *zo-1*, *muc2*, and *muc5* were investigated (Figure 3A). In the DEL-L group, immersion with DEL resulted in a significant reduction of *occludin* and *claudin12* mRNA expression in the gill of crucian carp (*p* < 0.05). Conversely, immersion with DEL led to a significant increase in *muc5* mRNA expression in the DEL-H group (*p* < 0.05). Although there was a trend towards decreased expression of other tight junction-related genes (*zo-1* and *muc2*), no statistically significant differences were observed when compared to the control group (*p* > 0.05).

#### 3.2.2. Expression of Apoptosis- and Autophagy-Related Genes

To analyze the effects of immersion with DEL on apoptosis and autophagy, the mRNA levels of caspase 3, caspase 8, caspase 9, bcl2, bax, beclin-1, atg 5, and atg 12 were investigated (Figure 3A). In the DEL-L group, immersion with DEL significantly upregulated the mRNA expression of beclin-1 (approximately 1.51-fold) in crucian carp gill (*p* < 0.05). In the DEL-H group, caspase 3 mRNA showed marked upregulation (4.87-fold, *p* < 0.05), and atg 5 mRNA followed a similar pattern in that a significant increase (approximately 6.70-fold) was observed compared to control group. In addition, the caspase 8, bax, and beclin-1 mRNA were slightly upregulated by 151%, 173%, and 169% in the DEL-H groups. No significant difference was observed in the mRNA expression of caspase 8, bcl2, and atg12 in the gills following immersion challenge (*p* > 0.05).

#### 3.2.3. Expression of Immune-Related Genes

To analyze the effects of immersion with DEL on immune response, the mRNA levels of Ig M, Ig T, lzm, il-1β, il-6, il-8, il-10, tnfα, ifnγ, tgfβ, tlr4, myd88, nf-kb, and ikba were investigated (Figure 3A). In the DEL-L group, immersion with DEL remarkably upregulated the mRNA level of lzm (approximately 2.29-fold), nf-kb (approximately 2.43-fold), and ikba (approximately 1.77-fold) in crucian carp gill (*p* < 0.05) while it downregulated the Ig T mRNA level (approximately 0.58-fold, *p* < 0.05) compared with the control group. In the DEL-H group, immersion treatment significantly increased the mRNA level of lzm (6.26-fold), il-6 (2.02-fold), il-8 (5.49-fold), il-10 (2.64-fold), tnfα (3.68-fold), ifnγ (3.14-fold), tgfβ (1.72-fold), tlr4 (2.75-fold), myd88 (4.78-fold), and nf-kb (2.42-fold) in crucian carp gill (*p* < 0.05) but had no significant effect on the il-1β mRNA level (*p* > 0.05). Meanwhile, compared to the control group, the mRNA expression of Ig M was significantly downregulated in the DEL-H group (approximately 0.61-fold, *p* > 0.05).

### 3.3. TMT-Based Quantitative Proteomic Analysis

**Figure 4:**
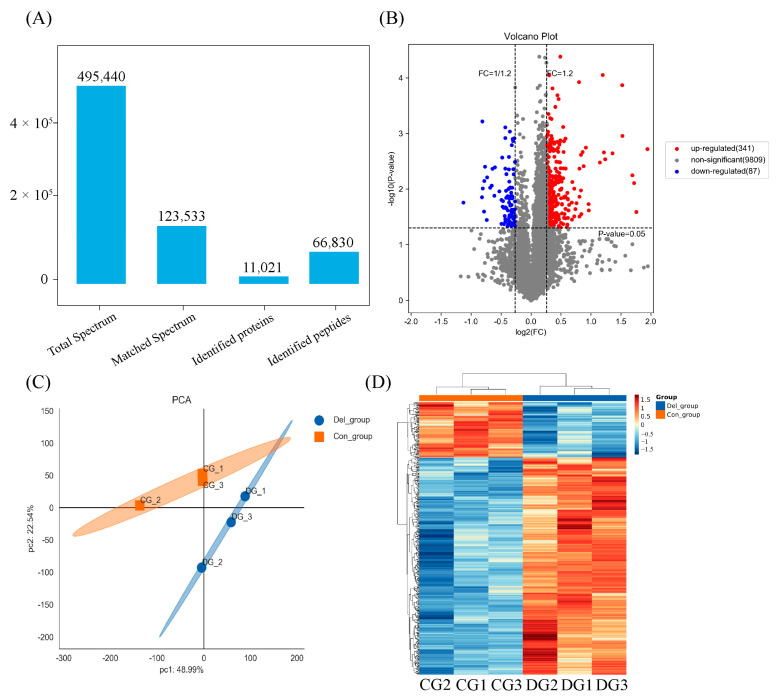
Statistics of TMT-based proteomic analysis. (**A**) Basic information statistics of proteome. (**B**) Volcano plots showing DAPs in the gill of crucian carp between the DEL-H and control groups. (**C**) The principal component analysis (PCA) in gill proteins between the DEL-H and the control groups. (**D**) A hierarchical cluster heatmap was generated to exhibit the relative expression of 428 DAPs in the gills between the DEL-H and the control groups. DAP: differentially abundant protein.

A total of 495,440 spectra were acquired through LC-MS/MS data analysis. Subsequently, Proteome Discoverer Software (Version 2.4.1.15, ThermoFisher Scientific, Waltham, MA, USA) was utilized to conduct database searches, resulting in the identification of 11,021 proteins, which were based on the crucian carp genome reference (Figure 4A). The reliability of the proteomic information was demonstrated by analyzing the dispersion of peptide lengths, the count of unique peptides, and the extent of protein coverage. These findings suggest that the identified proteins possess a significant degree of sequence coverage. After statistical analysis, a total of 428 DAPs were identified in the gills between the DEL-H and the control groups, including 341 upregulated DEGs and 87 downregulated DEGs (Figure 4B). In addition, the application of PCA revealed a distinct partitioning of gill proteins between the DEL-H and the control groups (Figure 4C). Following hierarchical clustering, a heatmap was generated to exhibit the relative expression of 428 DAPs in the gills, thereby uncovering significant variations between the DEL-H and the control groups (Figure 4D).

**Figure 5:**
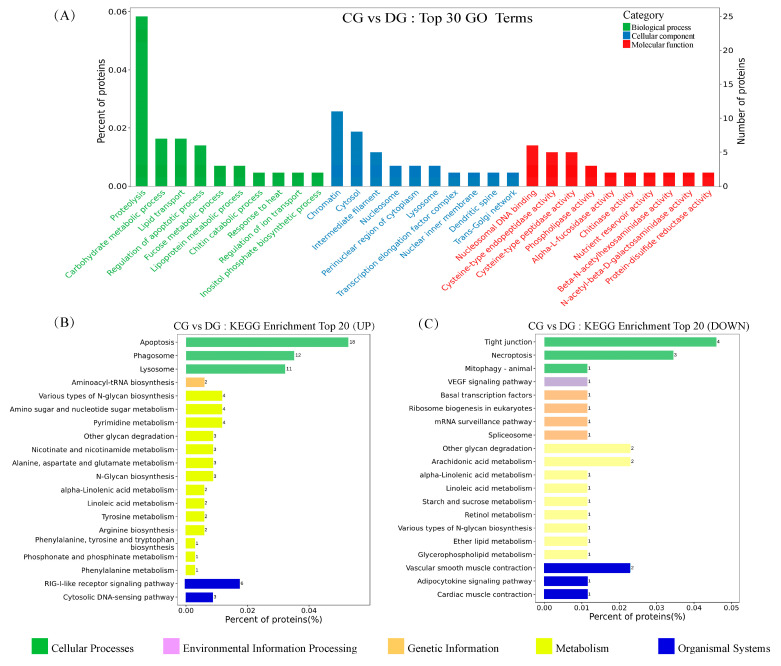
The results of KEGG pathway and enriched GO terms of functional enrichment analysis were visualized. (**A**) The top 30 most significant GO terms classified from 428 DEPs were identified using a histogram. (**B**) The top 20 upregulated KEGG enrichments of DEP in gills of crucian carp between the DEL-H and control groups. (**C**) The top 20 downregulated KEGG enrichments of DEP in gills of crucian carp between the DEL-H and control groups.

**Figure 6:**
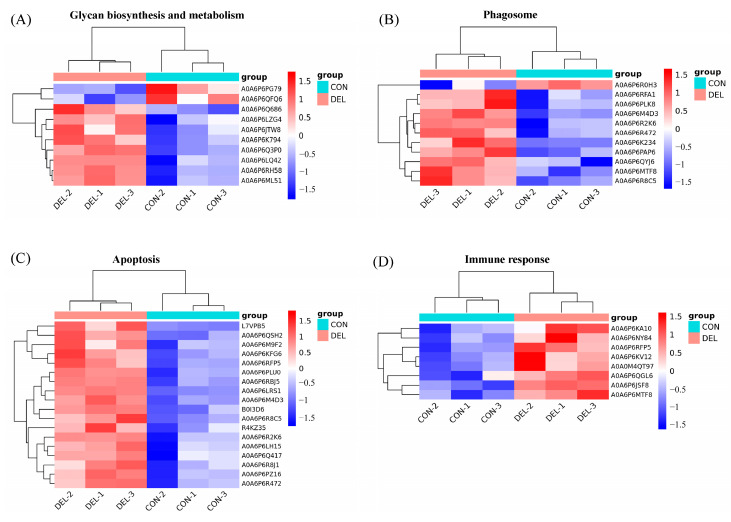
The heatmap shows the key signaling pathways were differentially regulated after DEL immersion, including glycan biosynthesis and metabolism (**A**), phagosome (**B**), apoptosis (**C**), and immune response (**D**).

**Figure 7:**
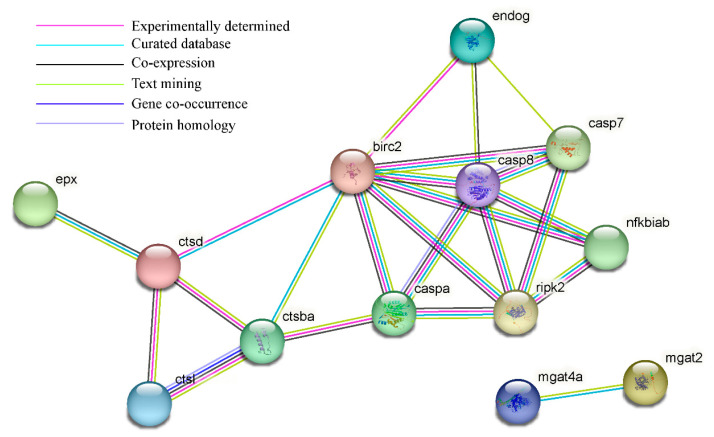
PPI analysis. Protein network of proteins regulated between glycan biosynthesis and metabolism, phagosome, apoptosis, and immune response-related gene expressed in the gill tissue. In the network, the representation of network nodes encompasses proteins in the form of splice isoforms or posttranslational modifications, wherein each node signifies the entirety of proteins generated by a singular gene locus responsible for protein coding. The edges within the network symbolize protein–protein associations, intended to convey specificity and significance, indicating that proteins collectively contribute to a shared function. It is important to note that these associations do not necessarily imply physical binding between proteins. Furthermore, the diverse colors of the edges correspond to distinct interactions.

According to GO analysis, 428 DAPs were clustered into three main categories, including biological processes (BP), cellular components (CC), and molecular functions (MF). For BP, proteolysis (GO:0006508), carbohydrate metabolic process (GO:0005975), and regulation of apoptotic process (GO:0042981) were more influenced by DEL immersion. For CC, chromatin (GO:0000785) and cytosol (GO:0005829) were the most represented terms. For MF, nucleosomal DNA binding (GO:0031492), cysteine-type endopeptidase activity (GO:0004197), and phospholipase activity (GO:0004620) were the most enriched terms (Table S2, Figure 5A). Meanwhile, KEGG enrichment was used to analyze the DAPs in the gills of crucian carp following DEL immersion. Of these, the three most notable KEGG pathways associated with upregulated DEPs were apoptosis (caua04210), phagosome (caua04145), and lysosome (caua04142), which exhibited significant enrichment based on cellular processes analysis. From a metabolism perspective, various types of N-glycan biosynthesis (caua00513), amino sugar and nucleotide sugar metabolism (caua00520), and N-glycan biosynthesis (caua00510) were significantly enriched in gill tissues (Table S3, Figure 5B). Meanwhile, among the downregulated DEP-enriched top KEGG pathways, many were involved in tight junction (caua04530), mitophagy (caua04137), and necroptosis (caua04217) signaling pathway (Table S4, Figure 5C). Importantly, numerous genes that were commonly associated with crucial pathways, such as glycan biosynthesis and metabolism, phagosome, apoptosis, and immune response, were found (Table S5). As shown in Figure 6, eight DEPs associated with glycan biosynthesis and metabolism (Galnt, Fuca2, LOC113119479, LOC113046654, mgat2, LOC113049817, mgat4a, and muc5ac) were significantly upregulated, and other two DEPs (Engase and Hexd) were significantly downregulated (Figure 6A). In addition, ten DEPs associated with phagosome (Tfrc, LOC113119101, LOC113102784, V-ATPase, Ctsl, LOC113070744, C3, Ctss, LOC113117520) were significantly upregulated, while the ras-related protein were downregulated (Figure 6B). Meanwhile, eighteen DEPs associated with apoptosis (Ire1, Ctsd, L7VPB5, Ctsx, Endog, Casp7, Ctsb, Casp1, Nfkbiab, Birc2, Ctsl, Capn2, Casp8, Ctss, and Prf1) and eight DEPs associated with immune response (Ripk2, Itgb, LOC113070744, LOC113092345, il-1Fm, Prf, Epx, and LZM) were significantly upregulated (Figure 6C,D). To investigate the interaction relationship in the gill of crucian carp following DEL immersion, a subset of DEPs associated with glycan biosynthesis and metabolism, phagosome, apoptosis, and immune response were selected for analysis using the STRING database to examine PPI networks (Figure 7). The functional interaction networks of Casp7, Casp8, Endog, Casp1, Nfkbiab, Birc2, Ctsl, Ctsd, Mgat2, Ripk2, and Mgat4a were identified as dynamic clusters for gill injury. These networks provided evidence of a correlation between gill injury and the caspase signaling pathway.

### 3.4. Gill Microbiota Analysis

In this study, the 12 gill samples of crucian carp yielded 957,216 raw reads, which underwent a stringent quality screening process resulting in 761,791 sequences and 4319 ASVs (Table S6). All raw sequences were deposited in the NCBI Sequence Read Archive under BioProject PRJNA977851. And rarefaction curves of sequences indicated that the sequencing depth was sufficient. 

**Figure 8:**
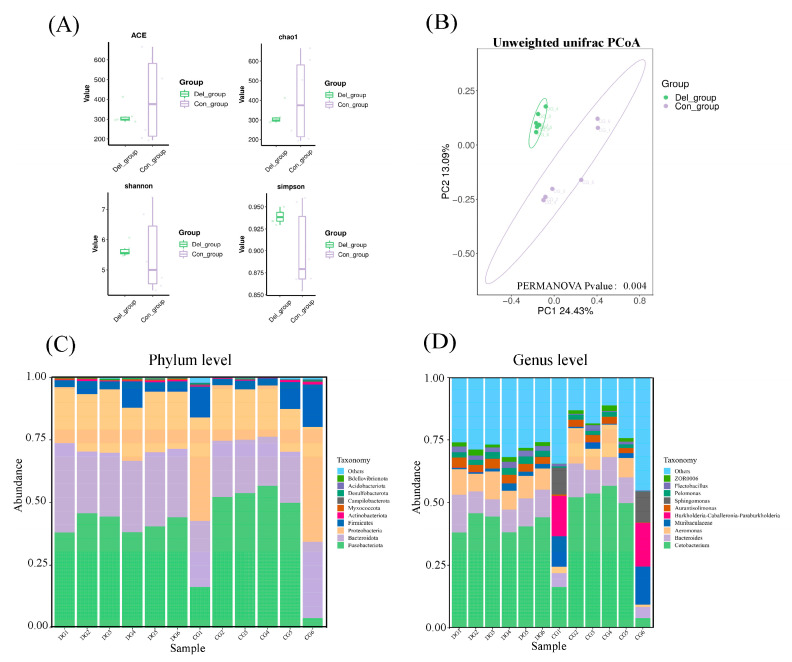
(**A**) The box diagram shows the alpha diversity of intestinal flora, including richness (ACE and chao1) and diversity (Shannon and Simpson); (**B**) beta diversity analysis was conducted using a PCoA diagram, which was generated using the weighted UniFrac distance. The percentage displayed on the diagram indicates the extent to which each principal component contributes to the variation observed among the samples; bacterial composition of the different communities in the gill of crucian carp after DEL immersion at the phylum level (**C**) and genus level (**D**).

**Figure 9:**
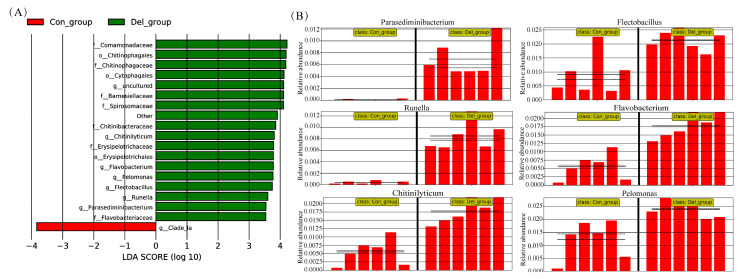
LEfSe analysis identifying taxonomic differences in the gill of crucian carp after DEL immersion. (**A**) Histogram showing LDA scores of the abundance of taxa; (**B**) relative abundance of key bacteria genus of gill symbiotic microorganisms including *Parasediminibacterium*, *Flectobacillus*, *Runella*, *Flavobacterium*, *Chitinilyticum*, and *Pelomonas*.

**Figure 10:**
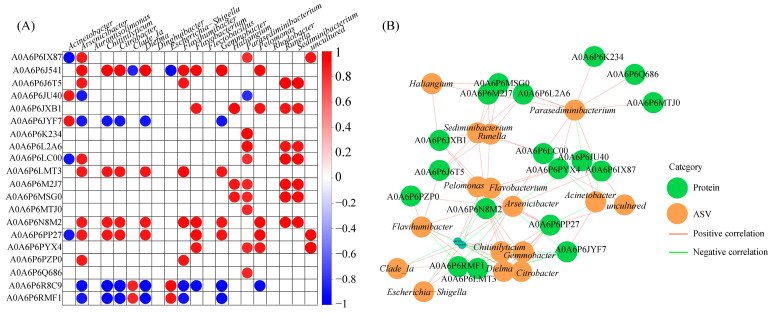
(**A**) Correlation analysis between the top 20 genus level of gill microbiota and DEPs. Red circles are positive correlations, while blue circles are negative correlations. (**B**) The relationships between DEPs and gill microbiota as revealed using associated network data analysis based on Spearman correlation analysis (*p* value ≤ 0.05).

The complexity of species diversity in the crucian carp gill was analyzed using alpha diversity. Our results showed that there were no statistically significant variations observed in the estimates of richness (ACE and Chao1) and diversity (shannon and simpson index) indices between the DEL and Con groups (Figure 8A). Moreover, it was found that the microbial communities from the gill samples were categorized into two distinct groups using principal coordinate analysis (PCoA) of unweighted uniFrac distances (Figure 8B). At the phylum level, Fusobacteriota was the most dominant phylum in all gill samples (40.31%), followed by Bacteroidota (25.96%), Proteobacteria (25.49%), and Firmicute (6.53%) (Figure 8C, Table S7). However, the relative abundance of the four phyla did not exhibit any noteworthy dissimilarities between the two groups. At the genus level, *Cetobacterium* (40.26%) and *Bacteroides* (9.70%) were the relatively abundant taxa between the DEL and Con groups (Figure 8D). In addition, LEfSe analysis identified six differentially abundant taxa as potential biomarkers in two different groups, including *Parasediminibacterium*, *Flectobacillus*, *Runella*, *Flavobacterium*, *Chitinilyticum*, and *Pelomonas* (Figure 9A). Compared with the Con group, there were remarkable increases in the relative abundances of all above bacteria in the crucian carp gills after DEL immersion (Figure 9B). Interestingly, Spearman analysis was conducted to evaluate the possible association between DEPs and gill microbiota genera, and the correlation was examined using the Wilcoxon test. As shown in Figure 10, the relative abundance of *Parasediminibacterium* had a strong positive correlation with the muc5AC (A0A6P6Q686). And the relative abundance of *Chitinilyticum* had a negative relation with NF-kappa-B inhibitor epsilon (A0A6P6RMF1) and cysteine-rich protein (A0A6P6R8C9), but a positive relation with quinone oxidoreductase PIG3 (A0A6P6LMT3).

## 4. Discussion

DEL is an insecticide widely applied to treat parasitic infections on the gills of fish species during freshwater culturing [5]. In practical application, the conventional anthelmintic concentration of DEL immersion on aquatic animals varies in different ranges such as 0.3–0.6 μg/L [16,17,18]. However, as a chemotherapy agent, DEL may have potential adverse effects on the external mucosa of fish when used for the treatment of parasitic diseases due to the external mucosa (such as gill and skin) having direct contact with the external environment [5,11]. Numerous studies have indicated that fish gills serve as a reliable indicator for monitoring the sublethal and long-term impacts of drug treatment and pollution [39,40]. Although previous studies on the toxicity of DEL to fish gills have predominantly focused on histopathological characteristics, as well as biochemical, physiological, and gene expression changes, there remains a dearth of comprehensive knowledge regarding the impact of insecticides on the immune system and microbiota in fish gills. In the present study, the histoarchitectural changes, gene expression, proteome, and external microbiota of gills were examined using HE and combined AB-PAS staining, TUNEL assays, qPCR analysis, TMT-based quantitative proteomic sequencing, and 16S rRNA analysis. Overall, our findings from all above experiments demonstrated conventional anthelmintic concentration of DEL immersion disorder in the gill immune microenvironment, including impaired barrier function, immunosuppression, and gill microflora dysbiosis. 

### 4.1. DEL Immersion Caused Destruction of Barrier Function in Gills

The epithelial cells of the gill serve as the first defense mechanism for fish against various environmental stresses, and its structural integrity is essential for preventing the infiltration of toxic substances and potential pathogens [1,30,36]. In the current study, microscopic observations of typical gill lesions are severe hyperplasia in the secondary lamella and apoptotic epithelial cells after DEL immersion, which were substantiated via HE and TUNEL assays. Gill epithelial hyperplasia is regarded as a key indicator for DEL toxicity in fish. Extensive research illustrates that exposure to DEL results in edema, hyperplasia, and hyperemia in the gill tissues of *Cyprinus carpio* [10], *Salmo trutta fario* [41], and *Carassius auratus* [11]. As expected, gills serve as a vital site for pesticide entry to the organism and exhibits a high absorption property of DEL, which is attributed to the high lipophilicity rate of DEL, thereby causing cell hyperplasia. Additionally, several studies have suggested that the presence of toxic substances, such as metallic nanoparticles, can interfere with the functional groups of amino acids, ultimately impacting the cell cycle and proliferation [38,42]. Similarly, in this trial, it was observed that numerous DEPs are involved in binding to nucleosomal DNA and amino acid metabolism were enriched in the GO and KEGG enrichment analyses. This finding implies that there may be an occurrence of mitotic instability. In turn, it is precisely this pathological change (hyperplasia) that thickens the gill epithelium, and it may be a protective response that reduces the absorption of harmful substances [43]. For example, exposure of Nile tilapia to elevated levels of ammonia resulted in the induction of hyperplasia in the gill filaments against the toxic effects of ammonia on the gills [44]. Regretfully, in routine histological analysis, it is often difficult to figure out the cell types involved in hyperplasia in gills. Further investigation is required to determine the specific mechanism responsible for this phenomenon by utilizing innovative biotechnological techniques such as single-cell RNA sequencing.

Our observation provides evidence for the hyperplasia of mucous cells within the gill epithelium. In addition, mucus is secreted from goblet cells and plays a crucial role in maintaining the physical and barrier function of gills [45], and fish have the ability to regulate mucus secretion as a mucosal barrier when subjected to stressful conditions [40]. The composition of fish mucus consists of many molecules, wherein the hyperglycosylated mucins serve as the basic skeleton. In teleost, the *muc5* genes exhibited predominant expression in the epidermis and gills, whereas the *muc2* genes displayed primary expression in the intestinal tissue [46]. The results of our qPCR analysis indicated that DEL immersion induces an increase in mucus secretion, resulting in the formation of a thicker mucus layer that restricts the movement of toxicants into host tissues [47]. Like this, exposure to herbicides and microplastics has been found to induce the secretion of mucus in an irregular manner, serving as a defensive mechanism to safeguard the epithelial mucosal surfaces [48,49]. Additionally, gill epithelial barriers rely on the cellular structures called tight junctions to enhance paracellular permeability, which selectively permits the passage of ions and soluble small molecules [36]. After DEL immersion, the transcription levels of tight junction genes including *occludin*, *claudin12*, *zo-1* were downregulated even though not significantly. Interestingly, the findings of our proteomic analysis revealed that immersion in DEL led to a reduction in the level of tight junction-related proteins, suggesting that posttranscriptional modifications play a role in the regulation of tight junctions. In fish, it has been demonstrated that exposure to chemicals leads to compromised tight junctions, resulting in heightened permeation across the gill epithelium, consequently elevating the toxicological risk [30,50,51]. As such, it is believed that the abnormal histological characteristics of gills are attributed to the destruction of gill barrier integrity, encompassing epitheliosis, mucus secretion, and epithelial cell junctions.

Apoptosis and autophagy are recognized as significant contributors to tissue injury in the mechanism of pesticide toxicity, as they represent two distinct modes of regulated cell death [12,52,53]. Numerous studies have found that DEL exposure can induce mitochondrial-mediated apoptosis by increasing the expression of inflammatory (caspase 1), apoptosis initiators (caspase 8), or executioners (caspases 3 and 7) [10,23]. In the current study, clear evidence of apoptosis was observed in the gills of crucian carp following immersion in DEL, as demonstrated through proteomic analysis and TUNEL assay. This is consistent with previous findings. In addition, our results showed that DEL immersion significantly decreased the expression of the p62 protein (sequestosome-1) in the gill, suggesting an enhancement in autophagic activity [54]. Syntaxin 17, another protein exhibiting upregulation and association with autophagy, was identified in our findings. To our knowledge, the involvement of syntaxin 17 in the fusion process between autophagosomes and lysosomes has been established [55]. Together with the results of qRT-PCR, we believe that DEL induces autophagy in gills through the regulation of autophagy marker genes. According to a recent study, the induction of autophagy through beclin-1 has been found to result in the generation of a thicker mucus layer in mice [47]. Consequently, it is postulated that exposure to pesticides might potentially influence the secretion of mucus in fish gills through a comparable mechanism. Overall, our results revealed that long-term DEL immersion results in disruption of tight junctions and the initiation of apoptosis in epithelial cells, ultimately resulting in the deterioration of gill epithelial integrity.

### 4.2. DEL Immersion Caused Disturbance of Mucosal Immune Response in Gills

As one of the mucosa-associated lymphoid tissues in teleost, the gill mucosa plays a crucial role as an immune barrier, encompassing both innate and adaptive immune responses [45]. Fish mucus contains many immunomodulatory molecules including lysozyme, immunoglobulin, proteases, complement system, and chemotactic cytokines [45]. In this experiment, the mRNA and protein levels of lysozyme were significantly upregulated after DEL immersion. This seems to contradict the results from previous studies [4,10]. This phenomenon could potentially be attributed to the stress effect, resulting in an upregulation of lysozyme expression. However, as exposure concentration increased, the influence of the dosage-dependent effect became more prominent, consequently leading to a downregulation of lysozyme expression. This is consistent with the trend of change in our previous results [11]. Previous studies have provided evidence for the existence of IgM and IgT in the serum and mucus of fish, where they play a vital role in both systemic and mucosal immunity [45]. The exposure of fish to insecticides results in the inhibition of IgM production in B-cells [6], and this study presents the initial findings that demonstrate the induction of a mucosal IgT response in crucian carp after DEL immersion. Complement factor C3 (C3) serves as the pivotal element within the complement system, acting as the point of convergence for both the lectin and alternative pathways of complement activation [56]. The findings of our study indicate that DEL immersion resulted in an increase in c3 protein levels, suggesting that low concentrations of DEL have the potential to induce an immunostimulatory effect in the gill tissue of fish. 

At the same time, previous research has demonstrated that environmental pollutants can induce inflammation responses in fish by disrupting the expression of immune-related cytokines [57]. Despite the absence of a substantial alteration in the mRNA expression of il-1β, the protein level of interleukin-1 family member A (il-1Fm) and nine inflammation genes exhibited a notable increase following DEL treatment, suggesting that immune cells (such as macrophages) swiftly generate these cytokines as factors of the acute phase response in response to tissue damage. In addition, the activation of the Toll-like receptor (tlr) signaling pathway has the potential to enhance the expression of inflammatory cytokines, thereby leading to the initiation of inflammatory responses [7]. Evidence has indicated that tlr4, a pattern recognition receptor, has the ability to be recognized and bound by oxidative specific epitopes that are induced by chemical poisons, thereby initiating the activation of the nuclear transcription factor nf-kb via the myd88 adaptor. This cascade ultimately culminates in the secretion of inflammatory cytokines [7,58]. In the present study, the activation of the tlr4 signaling pathway resulted in a significant increase in the expression of proinflammatory genes, as evidenced by correlation analyses of gene expression. Moreover, a notable increase in protein expression was observed for five cathepsin proteins (cathepsin D, X, B, L, S) following immersion in DEL. Cathepsins play a crucial role in multiple immune processes, including pathogen recognition and elimination, antigen processing and presentation, and apoptosis [59]. Additionally, as core mucus components, proteases have the ability to augment the synthesis of other innate immune components, such as complement systems and immunoglobulins [60]. Notably, the upregulation of CTSB, CTSL, and CTSZ in the gill tissue of crucian carp was found to be significantly enhanced in response to saline–alkaline stress, subsequently leading to an elevation in lysosomal membrane permeability [61]. Similarly, it has been observed that DEL exposure triggers apoptosis by upregulating CTSL in the kidney of goldfish [62]. Perforin (Prf) is a protein found within the cytoplasmic granules of CD8+ cytotoxic T lymphocytes in fish, where it is released to create pores on the membranes of target cells [63]. In the current experiment, these findings demonstrated that immersion in DEL induces cell apoptosis, subsequently leading to inflammation. In all, combined with the results of qPCR and proteomic analysis, we suppose that conventional anthelmintic concentration of DEL immersion caused immune disorders and inflammation in crucian carp gills.

### 4.3. DEL Immersion Caused Disorder of Commensal Flora in Gills

The function of fish gill mucosal surfaces is a distinct ecological niche for the recruitment and establishment of bacteria capable of colonizing and enduring on the mucosal surface [64]. Gill microbiota is an integral part of maintaining gill health, but the normal bacterial community of gill is influenced by the extensive application of antiparasitics and disinfectants [39,40,64]. The gill microbial community is highly susceptible to external environmental stressors as a result of direct exposure to the external environment. In this study, the richness in the DEL group was lower than that in the Con group even though not significantly. Importantly, PCoA analysis supported that there was a significant change in bacterial community species in the gill of crucian carp, suggesting an imbalance in the gill microbial community inducted by DEL immersion.

In accordance with previous research, the microbial composition within the gill of freshwater fish is commonly dominated by phyla Fusobacteriota, Bacteroidota, and Proteobacteria [65,66]. In the present study, while no significant disparity was observed in the dominant bacterial communities at the phylum level between the two groups, a noteworthy increase was identified in the predictive indicator bacteria *Parasediminibacterium*, *Flectobacillus*, *Runella*, *Flavobacterium*, *Chitinilyticum*, and *Pelomonas* within the DEL groups. These results indicate that DEL immersion had a more pronounced effect on the composition of bacterial genera with lesser abundances, rather than impacting the dominant bacterial families. Based on our findings, it was observed that there was a positive correlation between the abundance of *Parasediminibacterium* and the level of muc5ac protein. These data indicate the intrinsic effect of commensal bacteria on mucus secretion. Furthermore, *Flectobacillus* major, which was identified as a prominent species in both the skin and gills of trout during a microbiome survey, has been demonstrated to selectively regulate the expression and secretion of gill IgT, govern B cell populations, and impact the proliferation of other commensal bacteria within trout [67]. In addition, the analysis of the genome of a selected species of *Runella* revealed the presence of numerous genes linked to multi-drug efflux pumps, which potentially enable the removal of xenobiotics. This characteristic may contribute to the observed augmented abundance of the species following treatment with AgNPs [68]. Therefore, it was deduced that the presence of gill mucosa in fish served as a protective mechanism against DEL-induced toxicity, as it facilitated the proliferation of specific bacterial groups. Additionally, *Flavobacterium* has gained global recognition as a pathogenic agent in freshwater fish, capable of adhering to gill tissue and inducing gill rot. For instance, infections caused by *Flavobacterium* columnare manifest in skin lesions, fin rot, gill necrosis, and significantly elevated mortality rates [45]. Hence, the present study establishes a correlation between the higher abundance of these bacteria and immune dysfunction. Consequently, the findings suggest that DEL immersion can render the gill tissue barrier of fish more susceptible to bacterial infections. Similar findings indicated that the immune system of Chinese rare minnows was significantly suppressed and their susceptibility to *Pseudomonas fluorescens* infection was heightened after exposure to DEL [7]. Moreover, the chitinolytic bacterial species, known as the genus *Chitinilyticum*, was discovered in water samples collected from shrimp ponds. This bacterial genus has the potential to cause detrimental effects on shrimp health, specifically by inducing lesions on their chitinous exoskeleton [69]. Despite the scarcity of research concerning the involvement of *Chitinilyticum* in the external microbiomes of fish, our correlation analysis indicates that these bacteria may potentially undermine immune defenses. In addition, bacteria associated with disease biomarkers such as *Pelomonas* have been detected in the DEL group, indicating that DEL immersion directly affects the health of fish gills [70]. Therefore, DEL immersion may result in an increase in potentially harmful bacteria, consequently impairing the regular functioning of the immune system and disrupting the gill microenvironment of crucian carp, ultimately posing a risk to the overall health of the host.

## 5. Conclusions

In conclusion, the findings of this study offer innovative perspectives on the gill toxicity caused by the practical implementation of DEL in aquaculture using HE and combined AB-PAS staining, TUNEL assays, qPCR analysis, TMT-based quantitative proteomic sequencing, and 16S rRNA analysis. The immersion of crucian carp in a conventional anthelmintic concentration of DEL has been found to disrupt the mucosal barrier and immune system in gill tissue. Additionally, DEL immersion also leads to a disturbance in the commensal flora of the gills and an increase in potentially harmful bacteria. Consequently, the immune system’s normal operation is compromised, leading to a disturbance in the gill microenvironment of crucian carp. However, there are some limitations to our research. For example, further research should be conducted to explore the regulatory mechanism of DEL toxicity by utilizing more strict techniques such as single-cell RNA sequencing, semithin section, and possibly ultrastructure. The implications of the findings presented in this study are of great significance in advancing our understanding of the gill toxicity of DEL, thereby emphasizing the necessity of formulating strategies to alleviate the detrimental impacts of DEL on fish and other aquatic organisms.

## Data Availability

Data will be made available upon request.

## Supplementary Materials

The following supporting information can be downloaded at: https://www.mdpi.com/article/10.3390/toxics11090743/s1, Figure S1: The number of mucous cells in gills filament of crucian carp. Different capital letters indicate significant differences between different groups (*p* < 0.05). The values are expressed as the means ± SD. Error bars represent standard deviations of 10 replicates: Figure S2. The apoptosis rate was calculated using ImageJ software in the control, DEL-L, and DEL-H groups after DEL immersion. Different capital letters indicate significant differences between different groups (*p* < 0.05). The values are expressed as the means ± SD. Error bars represent standard deviations of 5 replicates; Table S1. Sequence of primer pairs used in the real-time quantitative PCR reaction; Table S2. Enriched GO terms of DAPs in gill of crucian carp between DEL-H and CON group. Table S3. Upregulated Enriched KEGG pathway terms of DEPs in the gills of crucian carp between DEL-H and CON group; Table S4. Downregulated Enriched KEGG pathway terms of DEPs in the gills of crucian carp between DEL-H and CON group; Table S5. List of DAPs related glycan biosynthesis and metabolism, phagosome, apoptosis, and immune response in gill between DEL-H and CON group of crucian carp; Table S6. Quality control and preprocessing of the original data from 16S rRNA sequencing; Table S7. The microbial composition in the gills of crucian carp after exposure to DEL immersion at the phylum level.
