# Supplementary material for: Conventional Anthelmintic Concentration of Deltamethrin Immersion Disorder in the Gill Immune Responses of Crucian Carp
Source: Toxics. 2023 Aug 31;11(9):743. doi: 10.3390/toxics11090743 (PMC10534886; doi:10.3390/toxics11090743)
Supplement: Supplementary file 1 [file toxics-11-00743-s001.zip › Supplementary material-figure S1-2.pdf]

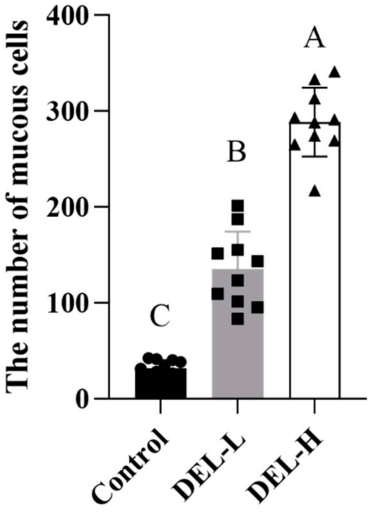

Figure S1. The number of mucous cells in gills filament of crucian carp. Different capital letters indicate significant differences between different groups ($P < 0.05$). The values are expressed as the means ± SD. Error bars represent standard deviations of 10 replicates.

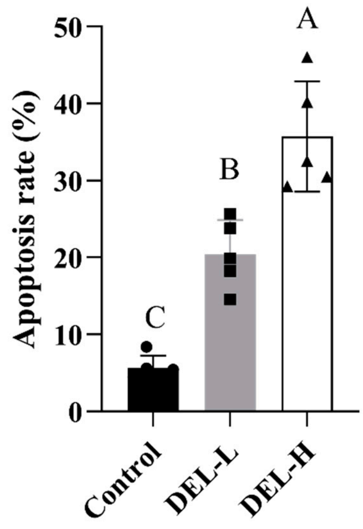

Figure S2. The apoptosis rate was calculated using ImageJ software in the control, DEL-L, and DEL-H groups after DEL immersion. Different capital letters indicate significant differences between different groups ($P < 0.05$). The values are expressed as the means ± SD. Error bars represent standard deviations of 5 replicates.
