# Supplementary material for: Conventional Anthelmintic Concentration of Deltamethrin Immersion Disorder in the Gill Immune Responses of Crucian Carp
Source: Toxics. 2023 Aug 31;11(9):743. doi: 10.3390/toxics11090743 (PMC10534886; doi:10.3390/toxics11090743)
Supplement: Supplementary file 1 [file toxics-11-00743-s001.zip › Supplementary material-table S1-7.pdf]

Table S1 Sequence of primer pairs used in the real-time quantitative PCR reaction.

Gene	Forward primer (5'-3')	Reverse primer (5'-3')	Accession number
Ig M	AGCTCAACCATCTGCACCAA	ATGTAAGCGAGTCCGCAGGT	GU563726.1
Ig T	ATTCATTGTCAGACCTTCACTCAG	GCAGTGTCTTCAGTCTTGAGGCT	MN170744.1
LZM	TGTGTCTGATGTGGCTGTGC	TGCACACATAGTTGCCAAGTGA	KJ703111.1
IL-1β	GATGCGCTGCTCAGCTTCT	AGTGGGTGCTACATTAACCATACG	AB757758.1
IL-6	CTGCCTGTCTCAGAGATCACAAGC	GCCGCAGACTATGCCGAAGAAG	AY102632.1
IL-8	GGTGTAGATCCACGCTGTCG	GTGAGGGCTAGGAGGGTAGAG	KC184490.1
IL-10	AGCCATGGGAGAGCTTGATA	ATGATGACGTGCAAGCGTT	HQ259106.1
TNFα	CATTCCTACGGATGGCATTTACTT	CCTCAGGAATGTCAGTCTTGCAT	EU069818.1
IFNγ	GAAACCCTATGGGCGATCAA	GTAGACACGCTTCAGCTCAAACA	EU909368.1
TGFβ	GTACACTACGGCGGAGGATTG	CGCTTCGATTCGCTTTCTCT	EU086521.1
TLR4	AAGTCCATCGCCTCCAACAT	AGCGACACCAGGCACTATCA	KT966378.1
MyD88	ACAGACAGAGCAAAAGTGACCA	GCCTTCCTCCTGATCCCAAC	MK246404.1
NF-κB	GCCACTAAATCCACCACATC	AACCCAAGCAGTTCACATACA	KM393205.1
IκBa	TTGCGAATCCAAAGGGGACA	TCTGTGATGACGGCGAGATG	XM_026291433.1
Occludin	AGTCCTCAGTTCCTGGATGCC	GTCCAGTAGCCGTTTCCTCTC	XM_026220502.1
Claudin12	ACTCCAGCCATGTCTTGTCG	AGCAGACCAGCAGGTAAACA	HQ656015.1
ZO-1	CGCCACACTTCTGACTACGA	TTATACATCTGCTGAGGCGCTG	XM_026267375.1
MUC2	CGACAATGCACTCATGCTGG	ACTTGTGGTTGGACCTGAGC	GH675184.1
MUC5AC	CGACAATGAAATCGTCCCGC	TTTCCGAAGACCACTGAGCC	GSE197741
Caspase 1	AAACCCAAGATCATCATCATCCA	CAGGGCATCAGCCTCTAAGTTGT	XM_026283617.1
Caspase 3	ATCATGACCAGGGTCAACCA	TACATCTCTTTGGTGAGCAT	XM_026266756.1
Caspase 8	TGTGAATCTTCCAAAGGCAAA	CTGTATCCGCAACAACCGAG	XM_026214093.1
Caspase 9	ATCACAAACTACCTCAACGG	CCTCCACAGGCCTGGATGAA	XM_026241892.1

Bcl2	AAAGGATGTACCAGCGCGAA	GGCTAAGAATCTGCGTTGCG	XM_026237836.1
Bax	ACCCCAGCCATAAACGTCTTGCG	GCCTTGATGACAAGCCGACAC	XM_026262399.1
Beclin-1	TGGAGAACTTGAGTCGCAGG	GCTGAGTGTCCAGATGGTCG	XM_026249455.1
Atg5	GCTCTTCCGACCAGTGTCTC	AGTTGTCTGGGTGGCTCAAG	XM_026284696.1
Atg12	GCTGTTGAAAGCAGTAGGTGATG	GGTCTGGTGATGGAGCAAATGAC	XM_026284438.1
β-actin	TTGAGCAGGAGATGGGAACCG	AGAGCCTCAGGGCAACGGAAA	AB039726.2

Table S2. Enriched GO terms of DAPs in gill of crucian carp between DEL-H and CON group.

Category	GO ID	Description	ListHits	PopHits	P-value
BP	GO:0006508	Proteolysis	25	1347	6.45E-08
BP	GO:0005975	Carbohydrate metabolic process	7	305	0.00117441
BP	GO:0006869	Lipid transport	7	3131	0.001361674
BP	GO:0042981	Regulation of apoptotic process	6	251	0.00389495
BP	GO:0006004	Fucose metabolic process	3	8	7.64E-06
CC	GO:0000785	Chromatin	11	37	4.93E-17
CC	GO:0005829	Cytosol	8	470	0.003468014
CC	GO:0005882	Intermediate filament	5	219	0.005978094
CC	GO:0000786	Nucleosome	3	105	0.017642492
CC	GO:0048471	Perinuclear region of cytoplasm	3	131	0.031251547
MF	GO:0031492	Nucleosomal DNA binding	6	13	3.15E-11
MF	GO:0004197	Cysteine-type endopeptidase activity	5	93	0.000131668
MF	GO:0008234	Cysteine-type peptidase activity	5	100	0.000185162
MF	GO:0004620	Phospholipase activity	3	41	0.001279881
MF	GO:0004560	Alpha-L-fucosidase activity	2	4	0.000160277

Table S3. Upregulated Enriched KEGG pathway terms of DEPs in the gills of crucian carp between DEL-H and CON group.

KEGG ID	Description	ListHits	PopHits	P-value
caua04210	Apoptosis	18	698	0.008746059
caua04145	Phagosome	12	628	0.162117989
caua04142	Lysosome	11	508	0.095827021
caua00970	Aminoacyl-tRNA biosynthesis	2	97	0.388092299
caua00513	Various types of N-glycan biosynthesis	4	155	0.167320759
caua00520	Amino sugar and nucleotide sugar metabolism	4	185	0.253052254
caua00240	Pyrimidine metabolism	4	190	0.268187107
caua00511	Other glycan degradation	3	42	0.020136877
caua00760	Nicotinate and nicotinamide metabolism	3	122	0.237888752
caua00250	Alanine, aspartate and glutamate metabolism	3	145	0.323871868
caua00510	N-Glycan biosynthesis	3	164	0.395211547
caua00592	alpha-Linolenic acid metabolism	2	72	0.262049317
caua00591	Linoleic acid metabolism	2	78	0.292759108
caua00350	Tyrosine metabolism	2	87	0.338443042
caua00220	Arginine biosynthesis	2	95	0.378283481
caua00400	Phenylalanine, tyrosine and tryptophan biosynthesis	1	15	0.188290566
caua00440	Phosphonate and phosphinate metabolism	1	25	0.293730939
caua00360	Phenylalanine metabolism	1	33	0.368158581
caua04622	RIG-I-like receptor signaling pathway	6	333	0.312887516
caua04623	Cytosolic DNA-sensing pathway	3	154	0.357802103

Table S4. Downregulated Enriched KEGG pathway terms of DEPs in the gills of crucian carp between DEL-H and CON group.

KEGG ID	Description	ListHits	PopHits	P-value
caua04530	Tight junction	4	1163	0.590639277
caua04217	Necroptosis	3	903	0.621174003
caua04137	Mitophagy - animal	1	352	0.713811754
caua04370	VEGF signaling pathway	1	357	0.718889403
caua03022	Basal transcription factors	1	119	0.343576444
caua03008	Ribosome biogenesis in eukaryotes	1	154	0.420242976
caua03015	mRNA surveillance pathway	1	371	0.732637871
caua03040	Spliceosome	1	501	0.832397619
caua00511	Other glycan degradation	2	42	0.009639265
caua00590	Arachidonic acid metabolism	2	166	0.116208944
caua00592	alpha-Linolenic acid metabolism	1	72	0.224655949
caua00591	Linoleic acid metabolism	1	78	0.240948822
caua00500	Starch and sucrose metabolism	1	96	0.287823989
caua00830	Retinol metabolism	1	147	0.405653364
caua00513	Various types of N-glycan biosynthesis	1	155	0.422298098
caua00565	Ether lipid metabolism	1	87	0.444434583
caua00564	Glycerophospholipid metabolism	1	87	0.794986629
caua04270	Vascular smooth muscle contraction	2	707	0.715752873
caua04920	Adipocytokine signaling pathway	1	377	0.738324594
caua04260	Cardiac muscle contraction	1	87	0.834795345

Table S5. List of DAPs related glycan biosynthesis and metabolism, phagosome, apoptosis, and immune response in gill between DEL-H and CON group of crucian carp.

Accession ID	Protein name	Description	Fold change	P-value
		**Glycan biosynthesis and metabolism**		
A0A6P6LZG4	LOC113063784	Polypeptide N-acetylgalactosaminyltransferase, Galnt	1.212389381	0.04481102
A0A6P6LQ42	fuca2	Alpha-L-fucosidase, Fuca2	1.293195719	0.011965774
A0A6P6RH58	LOC113119479		1.201834862	0.005142908
A0A6P6JTW8	LOC113046654		1.242990654	0.036331812
A0A6P6PG79	engase	Mannosyl-glycoprotein endo-beta-N-acetylglucosaminidase, Engase	0.82094082	0.03141198
A0A6P6QFQ6	hexd	Beta-N-acetylhexosaminidase, Hexd	0.79342302	0.04015537
A0A6P6ML51	LOC113068535	Alpha-1,6-mannosyl-glycoprotein2-beta-N-acetylglucosaminyltransferase, Mgat2	1.226345083	0.008494647
A0A6P6K794	LOC113049817	Alpha-mannosidase	1.252627628	0.011214374
A0A6P6Q3P0	LOC113108895	Alpha-1,3-mannosyl-glycoprotein 4-beta-N-acetylglucosaminyltransferase, Mgat4a	1.366863905	0.001821178
A0A6P6Q686	LOC113109550	mucin-5AC, Muc5ac	1.207873436	0.011455365
		**Apoptosis**		
A0A6P6KFG6	LOC113050786	serine/threonine-protein kinase/endoribonuclease, Ire-1	1.230026013	0.01054925
A0A6P6LRS1	LOC113061519	Cathepsin D, Ctsd	1.404809619	4.17274E-05
L7VPB5		Tubulin alpha chain	1.613943355	0.007426932
A0A6P6PLU0	LOC113105100	Cathepsin X, Ctsx	1.339181287	0.003231537
A0A6P6PZ16	endog	Endonuclease, Endog	1.226800297	0.014737371
A0A6P6QSH2	LOC113113788	caspase-7, Casp7	1.204704153	0.002422476
A0A6P6R8J1	LOC113117368	Cathepsin B, Ctsb	1.208317998	0.029767602
A0A6P6RBJ5	LOC113118288	Cathepsin D, Ctsd	1.20146789	0.00154806
A0A6P6LH15	LOC113059546	caspase-1, Casp1	1.26805293	0.026775862
R4KZ35	nfkbiab	Nuclear factor of kappa light polypeptide alpha b, Nfkbiab	1.311633282	0.027722582
B0I3D6	birc2	Baculoviral IAP repeat-containing 2, Birc2	1.294837476	0.00206265

A0A6P6M4D3	LOC113064852	cathepsin L1, Ctsl	1.292701567	0.001917241
A0A6P6M9F2	LOC113065999	calpain-2, Capn2	1.375692795	0.028984183
A0A6P6Q417	LOC113108941	caspase-8, Casp8	1.271866717	0.041043323
A0A6P6R2K6	LOC113116111	cathepsin S, Ctss	1.25611132	0.010746286
A0A6P6R472	LOC113116109	cathepsin S, Ctss	1.32738557	0.013781916
A0A6P6R8C5	LOC113117520	cathepsin L1, Ctsl	1.321083172	0.008196423
A0A6P6RFP5	LOC113119212	perforin-1, Prf1	1.232142857	0.006895602
		**Phagosome**		
A0A6P6QYJ6	LOC113115164	Transferrin receptor protein, Tfrc	1.433090024	0.018525936
A0A6P6RFA1	LOC113119101	H-2 class I histocompatibility antigen	1.27014756	0.030684646
A0A6P6PLK8	LOC113102784	antigen peptide transporter 2	1.415056361	0.040337564
A0A6P6K234	LOC113048548	V-type proton ATPase, V-ATPase	1.649448124	0.009833875
A0A6P6M4D3	LOC113064852	cathepsin L1, Ctsl	1.292701567	0.001917241
A0A6P6MTF8	LOC113070744	H-2 class II histocompatibility antigen	1.20146789	0.008208006
A0A6P6PAP6	LOC113097117	complement C3, C3	1.230483271	0.005067094
A0A6P6R0H3	LOC113115675	ras-related protein	0.764187004	0.024196396
A0A6P6R2K6	LOC113116111	cathepsin S	1.25611132	0.010746286
A0A6P6R472	LOC113116109	cathepsin S	1.32738557	0.013781916
A0A6P6R8C5	LOC113117520	cathepsin L1	1.321083172	0.008196423
		**Lysosome**		
A0A6P6LQ42	fuca2	Alpha-L-fucosidase	1.293195719	0.011965774
A0A6P6RH58	LOC113119479	Alpha-L-fucosidase	1.201834862	0.005142908
A0A6P6L6U0	LOC113056777	Legumain	1.254415633	0.02363488
A0A6P6LRS1	LOC113061519	Cathepsin D	1.404809619	4.17274E-05
A0A6P6PLU0	LOC113105100	Cathepsin X	1.3391812	0.00323153
A0A6P6R8J1	LOC113117368	Cathepsin B	1.208318	0.0297676

A0A6P6RBJ5	LOC113118288	Cathepsin D	1.20146789	0.00154806
A0A6P6M4D3	LOC113064852	cathepsin L1	1.292701567	0.001917241
A0A6P6R2K6	LOC113116111	cathepsin S	1.25611132	0.010746286
A0A6P6R472	LOC113116109	cathepsin S	1.32738557	0.013781916
A0A6P6R8C5	LOC113117520	cathepsin L1	1.321083172	0.008196423
		**Tight junction**		
A0A6P6Q8U6	LOC113110126	vasodilator-stimulated phosphoprotein-like isoform X1	0.581291173	0.025315087
A0A6P6REU8	LOC113118593	vasodilator-stimulated phosphoprotein-like isoform X4	0.572177102	0.009754143
A0A6P6LQY8	LOC113061731	src substrate cortactin-like isoform X1	0.815980629	0.031970667
A0A6P6QRZ9	LOC113113881	synaptopodin 2-like protein isoform X1	0.825121655	0.001219567
		**Autophagosome/ mitophagy**		
A0A6P6QPX5	LOC113113483	sequestosome-1, SQSTM1	0.783293698	0.000921516
A0A6P6LLE5	LOC113059854	syntaxin-17, STX17	1.223045572	0.022576693
		**Immune response**		
A0A6P6JSF8	LOC113046020	Receptor-interacting serine/threonine-protein kinase 2, Ripk2	1.224323322	0.000448693
A0A6P6KA10	LOC113050369	Integrin beta, Itgb	1.28745711	0.0385249
A0A6P6MTF8	LOC113070744	H-2 class II histocompatibility antigen	1.20146789	0.008208006
A0A6P6NY84	LOC113092345	V-type immunoglobulin domain-containing suppressor of T-cell activation	1.228369848	0.041780128
A0A6P6QGL6	LOC113111075	interleukin-1 family member A, IL-1Fm	1.248032971	0.027662906
A0A6P6RFP5	LOC113119212	perforin, Prf	1.232142857	0.006895602
A0A6P6KV12	LOC113054117	eosinophil peroxidase, Epx	1.333722287	0.031712149
A0A0M4QT97	LOC113066219	Lysozyme, LZM	1.34375	0.02676341

Table S6. Quality control and preprocessing of the original data from 16S rRNA sequencing.

Sample name	Raw reads	Filtered	Denoised	Merged	non_chimeric	ASV_counts
Con1	79392	68449	66773	59596	54615	606
Con2	78558	69459	68723	67306	65803	204
Con3	79775	70625	70040	69127	68144	245
Con4	78813	66694	65947	64259	62200	193
Con5	81571	70517	69533	66532	64692	504
Con6	78281	68537	66923	59448	53372	665
Del-H 1	81327	69985	68908	66344	63527	295
Del-H 2	78766	67391	66783	65712	64458	294
Del-H 3	79781	69397	68636	67339	65996	314
Del-H 4	78912	69615	68740	66275	64073	411
Del-H 5	80813	71334	70832	69895	68574	300
Del-H 6	81227	68836	68337	67459	66337	288

Table S7. The microbial composition in the gills of crucian carp after exposure to DEL immersion at the phylum level.

Phylum level	Relative Abundance (%)	
	Con group	Del group
Fusobacteriota	40.52 ± 3.05	41.87 ± 20.68
Bacteroidota	23.13 ± 3.60	28.47 ± 3.84
Proteobacteria	26.88 ± 1.34	23.17 ± 11.28
Firmicutes	7.74 ± 2.63	4.89 ± 3.51
Actinobacteriota	0.49 ± 0.25	0.51 ± 0.28
